# Investigations for Material Tracing in Selective Laser Sintering: Part ΙΙ: Validation of Modified Polymers as Marking Agents

**DOI:** 10.3390/ma16072631

**Published:** 2023-03-26

**Authors:** Tom Eggers, Frank von Lacroix, Martin Friedrich Goede, Christoph Persch, Werner Berlin, Klaus Dröder

**Affiliations:** 1 Volkswagen AG Wolfsburg, Berliner Ring 2, 38440 Wolfsburg, Germany; 2 Institute of Machine Tools and Production Technology, Technische Universität Braunschweig, Langer Kamp 19b, 38106 Braunschweig, Germany

**Keywords:** additive manufacturing, selective laser sintering, tracing, traceability, polymers, modified polymers, process optimization, process predictability, recycling, circular economy

## Abstract

Selective laser sintering (SLS) is currently in transition to the production of functional components. However, the ability to apply it is confronted with new requirements for reliability and reproducibility. Therefore, an in-depth understanding of aging processes in polymers is essential. Regarding material traceability as well as defective component identification with subsequent cause tracing, the application of a material-inherent marking technology represents a solution. SLS in combination with modified polymers as a marking technology proves to be an efficient opportunity to produce reproducible and high-quality components due to an increased understanding of the process. Based on a selection of modified polymers for use in SLS, which were characterized in part I of the study, this work focuses on the experimental validation of the result. The influence of modified polymers on materials and component properties and the SLS process’s influence on the traceability of modified polymers are examined. Intrinsic and extrinsic material properties as well as mechanical properties, surface quality and sinter density are analyzed. No discernible influences of the modified polymers on the investigated properties could be observed and the traceability of the modified polymers could also be confirmed in the aged powder and component using mass spectroscopy.

## 1. Introduction

The selective laser sintering (SLS) belongs to the additive manufacturing technologies. With the SLS technology, parts are produced by a layerwise deposition and targeted solidification of polymer powder by means of a thermal energy input. During sintering, the powder particles are fused together by the action of a laser beam [1,2,3,4,5,6,7]. Besides the printing process, the SLS process also involves pre- and postprocessing [2,4,5,8]. The polymer powder processing is affected by the properties of the material, which have been categorized as intrinsic and extrinsic properties in previous studies [5,8,9]. Furthermore, the printing result is affected by the selected SLS process parameters, for instance the intensity and speed of the laser [5]. In addition, the processability of the powder, primarily the flow behavior, is influenced by surrounding factors, such as the humidity and temperature [10,11,12]. In the SLS process, the building platform can be fully utilized, keeping minimum spaces between the consolidated parts, and the surrounding unconsolidated powder can be reused [1,2,3,4,5]. The material in SLS is subject to several aging phenomena and each of these has an impact on the specific material characteristics [4,13,14,15,16,17]. As a result, the stability of the SLS process as well as the quality and reproducibility of the mechanical component properties are affected [4,5,13,14,18,19,20,21]. To compensate these aging phenomena, a fraction-based mixing of recycled powder with 30% to 50% of new powder is common practice [4,5,13,19,20,21].

A fast and simple system to understand the aging state in polymers as well as the powder mixture composition and to adjust the SLS process parameters in order to decrease scattering is not yet available [13,22]. As a result, insufficient information is reflected in the final SLS part through defects in the surface, variations in the geometry, and an inadequate profile of the part’s properties [5,22]. In terms of material traceability and identifying nonconforming SLS parts with consequent cause tracing, the utilization of a marking technology for materials provides a possible solution. In part Ι of the study, a broad literature review was provided and modified polymers were ranked as the marking technology best suited for the SLS process [23]. A number of marking technologies was evaluated using a selection method. The selection method was based on a fusion of the utility analysis [24] and PROMETHEE method [25,26,27]. Modified polymers are encodable by targeted polymerization, thus enabling information storage at the molecular level [28]. SLS in combination with modified polymers as a marking technology has proven to be a suitable method for a reproducible and high-quality manufacturing based on a deeper knowledge of the process [29].

Therefore, the aim of the present study was the testing of modified polymers for use in SLS. The impact of the modified polymer on the properties of the unconsolidated and consolidated material and the influence of the SLS processing on the traceability of the modified polymer are investigated. Intrinsic and extrinsic properties of the material as well as mechanical properties of the component, surface quality, and sinter density are analyzed. The traceability of the modified polymer is measured by mass spectroscopy.

## 2. Materials and Methods

The materials and specimens for testing were stored and the test methods were applied under defined surrounding conditions of 50% relative humidity and a temperature of 23 °C. The storage of materials and specimens for testing was airtight and shielded from ultraviolet radiation.

### 2.1. Sinter Material

The used material for sintering in this study was a polyamide 12 material of the type LUVOSINT PA12 9270 BK manufactured by Lehmann & Voss & Co., KG (Hamburg, Germany). The selection of the specific sinter material resulted from the assumption that polyamide 12 was the main sinter material used in SLS [4,5,30,31]. In addition, this material was used in part Ι [23] of this study to select a suitable marking technology. Based on the manufacturer’s data, the selected polyamide 12 sinter material exhibited a specific density of 1.02 g/cm^3^ and bulk density of 0.40 g/cm^3^ [32].

### 2.2. Modified Polymer

The modified polymer was purchased from Polysecure GmbH (Freiburg, Germany) under the brand name POLTAG^®^ technology [29,33,34,35]. Further information on the marking agent are listed in part Ι [23] of the study.

### 2.3. Production of the Master Batch

A master batch was produced based on the used sinter material and the modified polymer. The production was carried out by a spray-drying process in which the modified polymer was applied to the sinter material under mixing. The concentration of the modified polymer in the master batch was 100 ppm.

### 2.4. Particle Analysis

A Camsizer XT particle analysis device from the manufacturer Retsch Technology GmbH (Haan, Germany) was applied for the particle shape and particle size distribution (PSD) analysis. The analysis was based on a dynamic image analysis as defined in ISO 13322-2:2021-12 [36]. In order to analyze the particles, five million recorded particles were scanned for each measurement. The method was in accordance with previous studies [37,38]. The particle aspect ratio and sphericity were provided as mean values of the measurement. Moreover, the PSD as well as the values of D10, D50, and D90 were known. No standard deviation was provided for this measurement method. The particle shape of the powder, which influences the flow behavior of the powder, was characterized by its aspect ratio and sphericity. As these are form factors, the maximum value for the aspect ratio and sphericity of the powder particle was a value of 1, also without a standard deviation [5,39].

### 2.5. Classification of Powder Density

The classification of powder density was based on the determination of the bulk and tap density. This method was also used in other studies [37,38]. Studies [40,41,42,43] reported that there was a correlation between the bulk density and flowability of the powder particles. In accordance with Spierings et al. [44], the Hausner factor [10,45,46] was not used in this study to determine the particle flow behavior, as it only indicates the ratio of the tap and bulk densities. A hopper supplied by the producer Landgraf Laborsysteme HLL GmbH (Langenhagen, Germany) was used to characterize the bulk density in accordance with DIN EN ISO 60:2000-01 [47]. For the determination of the bulk density, a material volume of 110 mL to 120 mL was taken. The powder ran through the hopper directly into a measuring cell. Through the ratio between the powder mass in the measuring cylinder and the volume of the measuring cylinder, the bulk density was determined. Using a tap volumeter device from the manufacturer Landgraf Laborsysteme HLL GmbH, the tap density was measured in accordance with DIN EN ISO 787-11:1995-10 [48]. A TR 120 drying oven from the manufacturer Nabertherm GmbH (Lilienthal, Germany) was used to dry the powder. The powder was stored at a temperature of 105 °C for a duration of 2 h. Then, the dried powder was cooled in a desiccator using silicate pellets to absorb moisture. Afterwards, a powder-measuring volume of 200 ± 10 mL was filled into the measuring cylinder. After 1250 camshaft rotations, the powder-measuring volume after the last compaction run was assumed as the ultimate volume. The calculation of the tap density was performed by the ratio of the powder mass in the measuring cylinder and the observed filling volume of the measuring cylinder. A EW 4200-2NM scale from the manufacturer KERN & Sohn GmbH (Balingen-Frommern, Germany) was used to quantify the powder mass in the measuring cylinder. Three calculations of the powder bulk and tap densities were taken. Then, the arithmetic mean value for both bulk and tap densities was calculated. This procedure deviated from the specifications in DIN EN ISO 60:2000-01 [47] and DIN EN ISO 787-11:1995-10 [48].

### 2.6. Scanning Electron Microscope

A Tescan Mira 3 scanning electron microscope (SEM) from the manufacturer Tescan GmbH (Dortmund, Germany) was used to analyze the particle shape. The SEM system takes images of the materials under investigation and analyzes them. An acceleration voltage of 15 kV was applied to the microscope. Secondary electrons were used for the detection. In order to ensure that the surface of the particles became electrically conductive, the investigated samples were initially sputtered for a duration of 40 s using gold [49]. This method was in accordance with previous studies [37,38].

### 2.7. Differential Scanning Calometry Testing

The differential scanning calometry (DSC) was carried out using a DSC-822 measuring device from the manufacturer Mettler Toledo (Gießen, Germany). The measurements were carried out under a nitrogen atmosphere. For each measurement, a sample weight of 10 ± 2 mg was selected. Furthermore, the cycles of heating and cooling were carried out in a temperature range between 25 °C and 230 °C using a heating/cooling rate of 10 °C/min. The DSC measurements were based on the DIN EN ISO 11357-1:2017-02 standard [50] and the method was in accordance with previous studies [37,38]. The thermal history of the material was first eliminated during the DSC measurement. As a result of the DSC measurements, among other data, both the melting temperature and crystallization temperature were output as mean values. Thereby, the DSC measurement did not contain any standard deviation.

### 2.8. Melt Flow Index Testing

For the testing of the melt flow index (MFI), the Mflow measuring device from the manufacturer Zwick/Roell GmbH & Co., KG (Ulm, Germany) was used. The MFI testing was in accordance with DIN EN ISO 1133-1:2012-03 [51]. A predrying of the powder was performed for a duration of 6 h at a temperature of 80 °C, which was in accordance with previous studies [37,38]. Therefore, a TR120 drying oven from the manufacturer Nabertherm GmbH was used. Before starting the test, the nozzle was cleaned using a cotton cloth and a cleaning tool. To determine the melt flow rate (MFR), a weight of 2.16 kg and a specimen mass of 4 g were used. The MFI testing for the used sinter materials was carried out at a temperature of 190 °C and a piston position of 50 mm. For the testing, three sections of 10 mm length of the extruded material were recorded. The mass of each of the three sections was measured with an AB-100 scale from the manufacturer PCE Deutschland GmbH (Meschede, Germany). Based on the extruded mass within the defined time interval, the MFR value was determined. This value was expressed in the unit g/10 min.

### 2.9. Mixing Technology

The sinter material and master batch were mixed using a Turbula T10B free-fall mixer from the manufacturer WAB AG (Muttenz, Switzerland). The used mixing device provides a mixing technology in which the particles are exposed to reduced mechanical forces. Furthermore, the mixer ensures that the powder is mixed as homogeneously as possible. The inside of the mixing container does not exhibit grinding bodies [40,52,53,54,55,56,57]. For the mixing process, a duration of mixing of 1 h and an intensity of mixing of 15 rpm were taken. Previous studies [37,38] confirmed the choice of mixing parameters. With these mixing parameters and the same experimental setup, a homogeneous and gentle mixing of the used polyamide 12 sinter material was recorded. For the powder mixture, mixing containers with a filling volume of 5 L were used. Thereby, a percentage level of filling of the mixing container of 75% was selected [38,58]. This filling level was confirmed by the previously mentioned studies. Concentrations of the modified polymer in the sinter material of 1 ppm and 20 ppm were produced.

### 2.10. Selective Laser Sintering Processing

**Figure 1:**
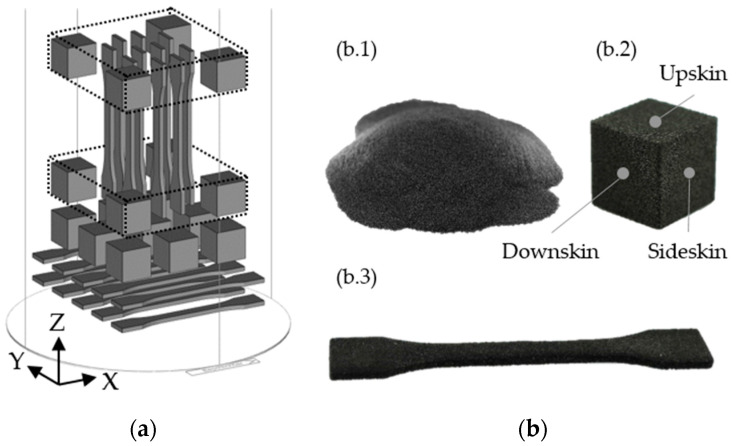
Design of the build job for the used SLS device [37]: (**a**) Alignment and placement of the test specimens in the building platform. The orientation of the XYZ-specimens is horizontal, and the orientation of the ZYX-specimens is vertical. The direction of coating occurs in the X-direction and the deposition of the layers occurs in the Z-direction. (**b**) Presentation of the used sinter material and test specimens: (b.1) powder in the printing as well as in the aged condition; (b.2) cube with investigated surface orientations; (b.3) tensile bar.

For the SLS processing, a Sintratec S2 SLS system from the manufacturer Sintratec AG (Brugg, Switzerland) was used. The operating principle was based on the processing of selective laser sintering polymers described in several studies [2,4,5]. The building platform was designed cylindrically and exhibited a usable diameter of 130 mm. The maximum working height of the building platform was 360 mm. The Sintratec S2 system includes a diode laser with a laser power of 10 W and a laser wavelength of 1064 nm. The diameter of the laser spot was 145 μm. According to the manufacturer’s specifications and a previous study [37], a 100% laser power was used for the sinter material at hand. During hatching, a laser scanning speed of 3.85 m/s was used. The layer offset was 90°. In contrast, the scanning speed for printing the boundary was reduced to 3.20 m/s. For the sinter material at hand, the temperature of the surface of the powder bed was heated up to 175 °C. The procedure was in accordance with a previous study [37], and no inert gas was applied throughout the sintering process, due to the selected SLS system. For the investigated sinter material, a layer thickness of 100 μm was taken. Prior to the SLS processing, a vacuum cleaner and wipes with isopropanol were used to clean the depowdering tools and the entire SLS system. Figure 1a shows the used standardized build job layout. Based on the used build job and a packing density of 3.3%, a quantity of new powder of 3.6 kg was provided for each printing process. This powder was called printing powder.

Table 1 lists the components of the build job illustrated in Figure 1b. Three printing processes of each type of powder mixture were carried out and evaluated to analyze the properties of the components. The arithmetic mean value of each investigation was calculated over three printing processes. Since polyamides have a glass transition temperature, depowdering was conducted with a brush, after cooling down to a powder bed core temperature of 40 °C [4,5]. The entire unsolidified powder from the print job was extracted by an NT 30/1 Te H vacuum cleaner from the producer Kärcher (Winnenden, Germany) and separated by a DLX MKII cyclone separator from the producer Dust Commander (Sélestat, France). All unsolidified powder was composed of the powder from the feeders, the overflow, and the building platform, and was henceforth defined as recycled, aged powder.

### 2.11. Tensile Test

To realize the tensile tests, a Zwick/Roell Z100 tensile testing machine from the manufacturer Zwick/Roell GmbH & Co., KG was used. To achieve a dry condition, the specimens (Table 1) were stored in a V0 400 drying vacuum oven from the manufacturer Memmert (Schwabach, Germany) for a duration of 95 h at a temperature of 60 °C. This procedure was in accordance with a previous study [37]. During drying, a vacuum level of 70 mbar was present. Drying was performed according to DIN EN ISO 16396-2 [60]. After drying, the specimens were placed in airtight containers together with silicate pellets to absorb moisture. The tensile tests in this study were realized within five days after drying of the specimens, which was in accordance with a previous study [37]. A makroXtens mechanical extensometer and two wedge clamping jaws designed for a normal force of up to 10 kN were used. In addition, an Xforce K load cell approved for the same load limit was applied. The testing machine was equipped with a testControl II control unit. The tensile test was performed in accordance with DIN EN ISO 527-1 [61]. While elongation at break and tensile strength were determined at a 50 mm/min test speed, the Young’s modulus was determined at a 1 mm/min test speed and the specimens were subjected to a preload of 0.1 MPa. The test speeds were in accordance with DIN EN ISO 16396-2 [60]. As defined in DIN EN ISO 527-1 [61], the Young’s modulus was determined as the secant modulus in the interval of elongation from 0.05% to 0.25%. For each investigated concentration, 30 tensile tests were evaluated (three build jobs with ten tensile bars in each direction).

### 2.12. Sinter Density

For the determination of the sinter density, the gravimetric density measurement was applied [5]. The geometric measurement of the specimens (Table 1, cubes ^2^) was performed with a VL-550 optical coordinate measuring system from the manufacturer Keyence Corp. (Neu-Ilsenburg, Germany). The selected settings on the measuring system are listed in Table 2 and are in accordance with a previous study [37].

The contours of the cube were recorded with an accuracy of ±10 μm using strip light projections. First, the specimens to be examined were digitized and captured as tessellated half-shells. Then, the volume of the specimens was specified by manually stitching the individual images together to form a closed body. Using an AB-100 scale from the manufacturer PCE Deutschland GmbH, the mass of the specimens was determined. The sinter density was calculated from the quotient of the mass and volume of the specimens.

### 2.13. Confocal Microscopy

For the determination of the surface roughness and digitization of the topography, a MarSurf CM mobile confocal microscope from the manufacturer NanoFocus AG (Oberhausen, Germany) was used. The measurements were performed in accordance with DIN EN ISO 4287:2010-07 [62] and DIN EN ISO 25178-1:2016-12 [63]. Three main orientations [1,5] (upskin, sideskin, downskin) of the specimens (Figure 1b) listed in Table 1 (cubes ^3^) were analyzed. As a result, it was possible to determine the influence of the orientation of printing on the respective roughness of the surface. The measuring field was located in the center of the surfaces of the cube. Using μsoft analysis extended software 7.4.8737, the created data were converted into information on the roughness and flatness. The partially direction-dependent roughness of the surfaces was mentioned in previous studies [1,5,64,65]. As a result, the surface parameters S_a_ and S_z_ were taken to evaluate the surface roughness of the specimens. The S_a_ value describes the average arithmetic height and the S_z_ value the maximum height of the surface. The selected settings on the used confocal microscope are listed in Table 3 and are in accordance with a previous study [37]. For each investigated concentration, 27 specimens were evaluated with respect to the three measurements surfaces (three build jobs with nine tensile bars).

### 2.14. Tandem Mass Spectroscopy

Tandem mass spectroscopy (MS/MS) was used to detect and sequence as well as measure the traceability of the modified polymer [28,66,67,68]. An API 4000 MS/MS device from AB SCIEX (Darmstadt, Germany) was used. Table 4 lists the different extraction methods for the investigated samples (Table 1). The analyte was transferred to the atmospheric pressure chemical ionization source using a Model 100 syringe pump from kdScientific Inc. (Holliston, MA, USA). The volume flow of the syringe pump amounted to 0.06 mL/h. Both detection and sequencing were measured for a duration of 5 min. Between the individual measurements, the MS/MS device was cleaned by injecting a liquid consisting of methanol and 3 mmol ammonium acetate for at least 10 min. Based on a python tool, the results of the MS/MS measurements were evaluated. The sum of the determined intensities was calculated within a measurement interval of ±1 Da for all mass numbers of the modified polymer. The modified polymer was considered to be detected if an intensity was determined for the mass number of the main mass as well as for the majority of the mass number of all sequences within the measurement interval and if the peaks were clearly visible.

## 3. Results

### 3.1. Influence on the Material Properties

In Table 5 the examined material properties for the sinter material and master batch are listed. Compared to the sinter material, the master batch exhibited an approximately 1.7% higher D10-value, an approximately 1.6% lower D50-value and a 1.0% lower D90-value. The master batch showed an approximately 0.4% higher sphericity and a 0.3% higher aspect ratio than the sinter material. There was no deviation in bulk density and tap density between the sinter material and master batch considering the standard deviation. Furthermore, the master batch had a 0.14% lower crystallization temperature and a 0.17% lower melting temperature than the sinter material. Taking into account the standard deviation, the MFR value of the two materials did not deviate from each other.

**Figure 2:**
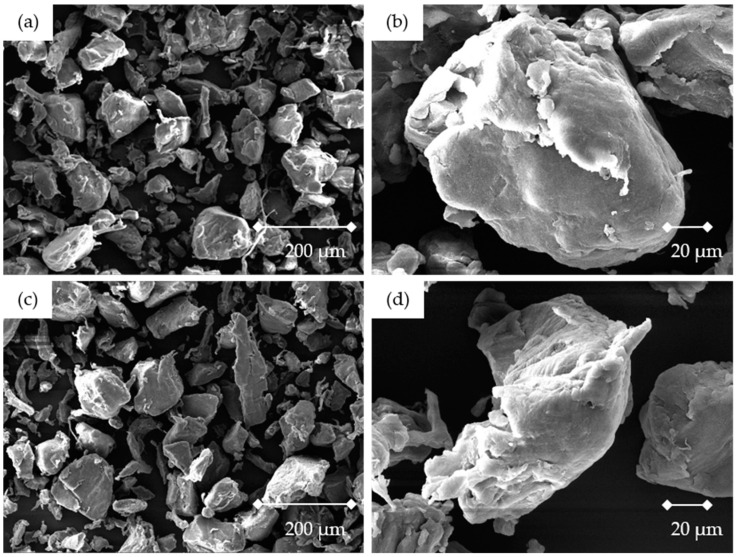
SEM images of the used materials: (**a**,**b**) Polyamide 12 sinter material at 168× and 828× magnification; (**c**,**d**) Master batch based on the polyamide 12 sinter material and the modified polymer at 168× and 824× magnification. Produced with a Tescan Mira 3 microscope.

In Figure 2 the SEM images of the used sinter material and master batch are presented. Two different magnifications of the powders were considered. A ragged, undefined particle shape was present in both the sinter material and master batch. In addition, there was an irregular particle size of the individual particles. The particle size was in accordance with the particle size distribution shown in Table 5. No qualitative difference between the two materials in particle shape or size was visible in the SEM images. The materials appeared visually black.

### 3.2. Influence on the Component Properties

**Figure 3:**
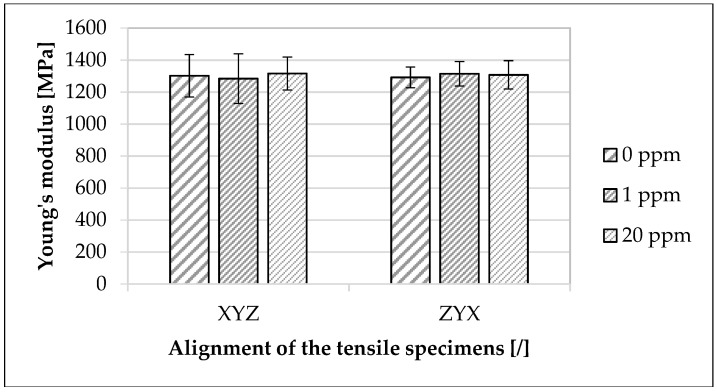

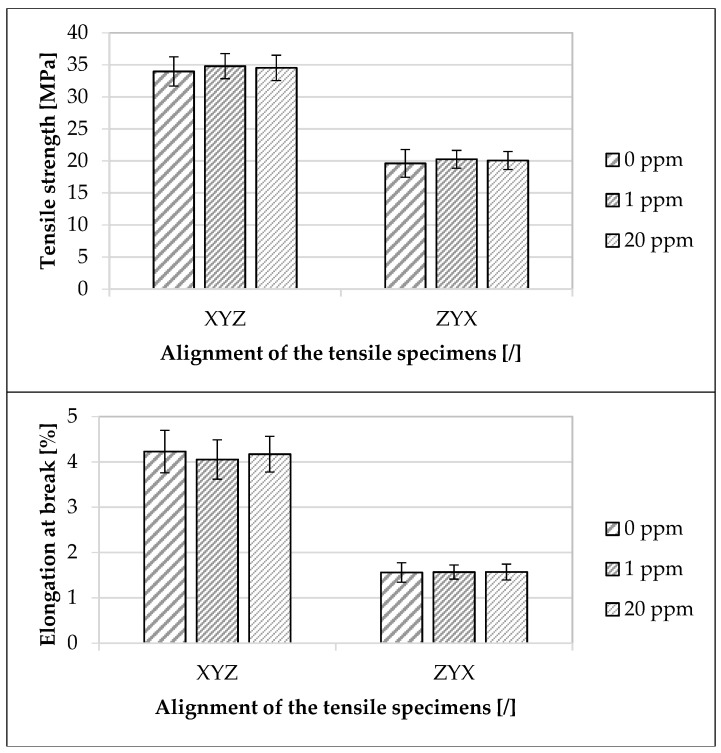
Influence of the used modified polymer on the investigated mechanical properties. The specimens were made of the used polyamide 12 sinter material. Concentrations of the used modified polymer of 0 ppm, 1 ppm, and 20 ppm and the XYZ- and ZYX-directions were examined. For each concentration and direction, 30 tensile tests were performed.

In Figure 3, the influence of the used modified polymer on the mechanical properties in the XYZ- and ZYX-directions depending on the concentration is presented. Regarding the mean value of Young’s modulus, from 0 ppm to 20 ppm, there was an increase of about 1.1% for the XYZ-direction and about 1.2% for the ZYX-direction. Regarding the mean value of tensile strength, there was an increase of about 1.7% in the XYZ-direction and about 2.4% in the ZYX-direction. The mean value of elongation at break decreased by about 1.4% in the XYZ-direction and increased by 0.6% in the ZYX-direction.

**Figure 4:**
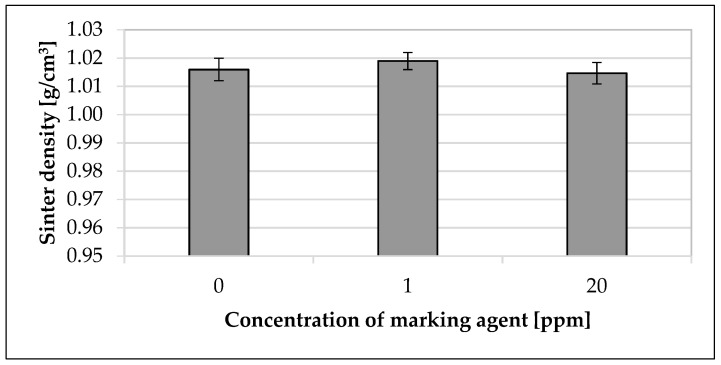
Influence of used modified polymer on the sinter density. The specimens were made of the used polyamide 12 sinter material. Concentrations of the used modified polymer of 0 ppm, 1 ppm, and 20 ppm were investigated.

In Figure 4, the influence of the modified polymer on the sinter density depending on the concentration of the modified polymer is presented. The mean value of the sinter density increased by 0.3% for 1 ppm and decreased by 0.1% for 20 ppm.

**Figure 5:**
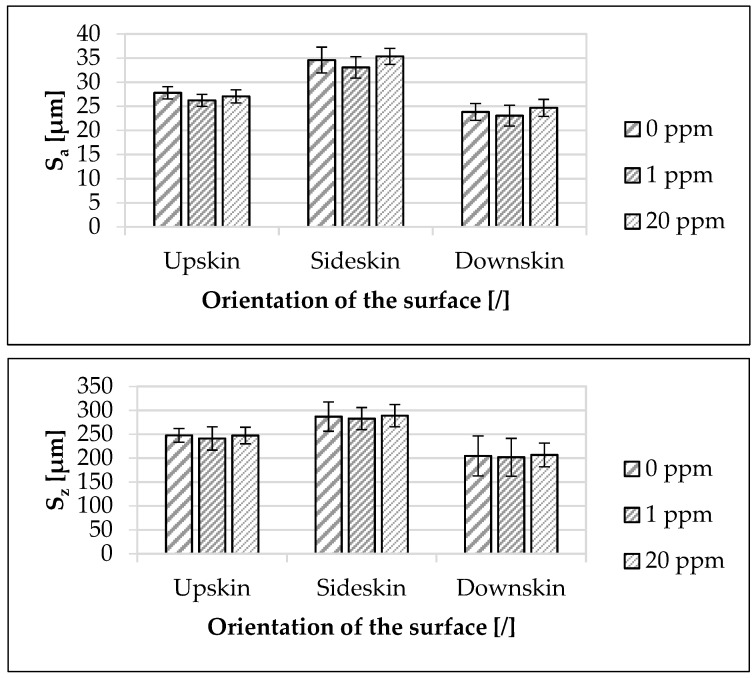
Influence of the used modified polymer on the surface roughness (S_a_ and S_z_). The specimens were made of the used polyamide 12 sinter material. Concentrations of the used modified polymer of 0 ppm, 1 ppm, and 20 ppm were investigated. Upskin, sideskin and downskin orientations were examined.

In Figure 5, the influence of the modified polymer on the surface roughness (upskin, sideskin, downskin) depending on the concentration of the modified polymer is presented. In relation to the upskin surface, the S_a_ mean value decreased by 2.6% from 0 ppm to 20 ppm. The mean value of the sideskin surface increased by 2.2% and the mean value of the downskin surface by 3.6%. Regarding the S_z_ mean value, the roughness of the upskin surface decreased by 0.2% from 0 ppm to 20 ppm. The mean value of the sideskin surface increased by 0.7% and the mean value of the downskin surface by 1.0%.

The deviations of the mean values of the investigated component properties depending on the concentration of the modified polymer were within the standard deviation in each case.

### 3.3. Traceability of the Marking Agent

**Figure 6:**
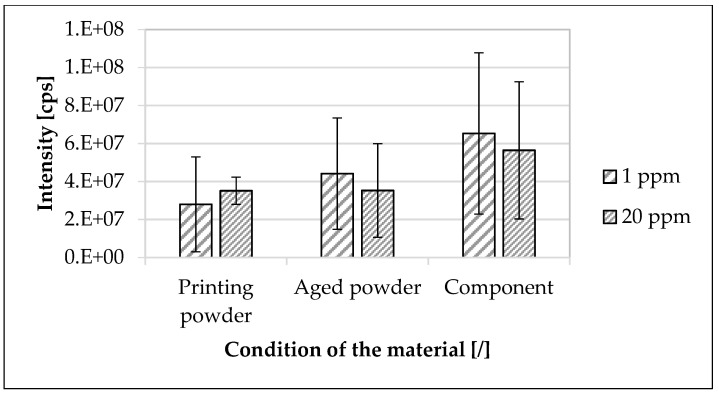
Traceability of the used modified polymer at different material conditions. The printing and used powders as well as the component were considered. The specimens were based on the used polyamide 12 sinter material. The arithmetic mean of the sum of the intensities within the considered interval of ±1 Da around the main molar mass of four MS/MS measurements each was calculated. Concentrations of the used modified polymer of 1 ppm and 20 ppm were considered.

**Figure 7:**
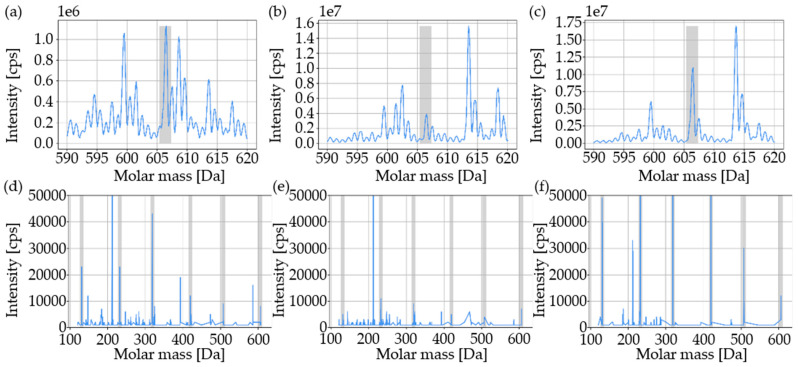
MS/MS measurements (detection and sequencing) of the material conditions for a 1 ppm concentration of the used modified polymer. Intensity is plotted over the molar mass: (**a**,**d**) printing powder; (**b**,**e**) aged powder; (**c**,**f**) component. The intervals in which the main masses and the individual sequences were detected are highlighted in gray. The specimens were based on the used polyamide 12 sinter material.

In Figure 6, the traceability of the modified polymer at different material conditions (printing and aged powders, component) is shown. The modified polymer was detectable at a concentration of 20 ppm as well as 1 ppm in the printing powder, aged powder, and in the component. In all material conditions, the main mass as well as all individual sequences were detectable (Figure 7).

No correlation between the concentration of the modified polymer and the mean value of the detected intensity of the main mass was apparent (Figure 6 and Figure 7). Taking into account the standard deviation, there was no change in intensity between printing and aged powders for both concentrations of the modified polymer. The mean value of the intensity of the modified polymer (1 ppm) in the components was approximately 133.6% higher than the mean value of the intensity of the modified polymer in the printing powder and approximately 48% higher than the mean value of the intensity of the modified polymer in the aged powder.

## 4. Discussion

Based on the broad literature review and the systematic choice of a suitable marking agent for an application in SLS in part Ι [23] of this study, part ΙΙ focused on the experimental validation of the result. In particular, the focus was on the influence of the modified polymer on the intrinsic and extrinsic material properties and mechanical component properties, surface quality, and sinter density as well as on the influence of the SLS process on the traceability of the modified polymer.

The investigated extrinsic and intrinsic material properties (Table 5) as well as the sinter density and mechanical component properties (Figure 3 and Figure 4) were not significantly influenced by the modified polymer. This observation was confirmed by various studies [5,8,69,70,71] describing the general material properties and component characteristics in SLS without using marking agents. Although the material properties of the sinter material and the master batch differed and the component properties increased slightly with an increasing concentration of the modified polymer, the change in the investigated properties was within the standard deviation of the mean values. Thus, the processing properties of the marked polyamide 12 sinter material remained the same as those of the unmarked material (Table 5). In particular, this was confirmed by the fact that the selected concentration of 100 ppm was already maximum and thus lower concentrations behaved analogously in the used sinter material. The marginal increase of the component properties with the increasing concentration referred to the corresponding mean values. Taking into account the minimum and maximum standard deviation, the change in component properties due to the addition of the modified polymer was negligible. In addition, minor changes in material charges between testing runs may have been the cause of the changes. The observation that the change in the investigated properties was within the standard deviation may be due to the fact that the selected concentrations of the modified polymer were too low to have any effect on the investigated material and component properties using the selected testing methods [72]. Furthermore, the possible influence of the modified polymer could be overlaid by other additives in the sinter material, such as antistatic agents, flow aids, or carbon black particles [14,73]. The chosen concentrations of the modified polymer resulted on the one hand from the simplified handling of the master batch to adjust to lower concentrations and on the other hand from the choice of a target concentration for the future application of the marking agent. The choice of concentration in the master batch was also in accordance with the manufacturer of the modified polymer. For future application of the marking agent in the SLS process and the stepwise coding of the sinter material regarding a tracer-based process optimization [23], the maximum concentration of the modified polymers in the used sinter material was up to 20 ppm. When using a concentration of the modified polymer of 1 ppm per printing process, a maximum concentration of the sum of different modified polymers of no more than 20 ppm could be expected after 20 cycles. For more than 20 cycles, the respective concentration of the specific modified polymer (initially 1 ppm) was so low, due to the usual refresh rates [4,5,13,19,20,21], that this concentration, as well as the amount of powder related to it, could be discounted. The concentration of 1 ppm was already sufficient to ensure a full functionality and traceability of the modified polymer in the used sinter material (Figure 6 and Figure 7). The chosen concentration of modified polymers in various materials was also confirmed by various studies [29,33,34,66,74]. The choice of higher concentrations of the modified polymer did not represent an added value in terms of functionality or traceability.

The investigations presented in Figure 3 showed a clear anisotropy of the specimens, which was confirmed by previous studies [4,5] that also recorded anisotropy as a characteristic of the SLS process. Thereby, the ZYX-specimens exhibited a lower elongation at break and tensile strength than the specimens in the XYZ-direction. The reason for the isotropic appearance of the Young’s modulus was the measurement method. As confirmed by previous studies [5,49], the Young’s modulus was determined in a deformation window wherein the anisotropic specimen behavior was not yet present in the result. Contrary to previous studies [4,64,65,75,76,77] where the downskin surface did not exhibit the lowest surface roughness, the downskin surface showed the lowest surface roughness for all concentrations of the modified polymer (Figure 5). According to these investigations, the sideskin surface exhibited the highest surface roughness. A previous study [37] indicated a similar behavior of the surface roughness as a function of the orientation of the measurement surface to the buildup direction for the used polyamide 12 sinter material but without using modified polymers. The reason for the deviation of the results from the observations of the previously mentioned studies could be due to the used process parameters and SLS equipment, as well as the used polyamide 12 sinter material. As a result, a planar powder bed surface was not already given during powder deposition [78,79]. Another possibility was mentioned in a previous study [37], which pointed out too short a dwell time of the powder application as a possible cause. As a result, the top layer was not completely consolidated when the next layer of powder was applied. This could result in powder particles being embedded in the component surface and affecting the surface roughness. Even though this observation was not explored further in this work, a similar behavior was discussed in a previous study [37].

The modified polymer was detectable in the printing powder as well as in the aged powder and component down to a concentration of 1 ppm (Figure 6 and Figure 7). Since there was no visible correlation between the concentration of the modified polymer and the detected mean value of the intensity of the main mass, only a qualitative evaluation was possible. The influence on the measurement was possibly due to the presence of carbon black particles in the used sinter material. The carbon black particles were dissolved out of the used sinter material during the extraction process (Table 4) and were deposited in the measuring chamber of the mass spectroscope during the MS/MS measurement. As a consequence, no clear quantitative determination could be made about the degree of homogeneous mixing of the used sinter material and master batch as well as the influence of the SLS process on the traceability of the used modified polymer. A further developed filtration technology could solve this phenomenon and enable a quantitative determination. Since the used modified polymer was reliably detectable in all material samples along the SLS process down to a concentration of 1 ppm, a sufficient dispersion could be assumed with the selected mixing parameters. Due to the manufacturing process of the used master batch, it could be assumed that all powder particles were marked. The reason for the higher intensity of the modified polymer in the component was possibly that the used sinter material and thus also the additives were bound and/or had evaporated. As a result, the measurement result was less strongly influenced than with the powders.

## 5. Conclusions

Ensuring traceability of materials in the SLS process and identifying defective components followed by tracing the cause can potentially be achieved by the application of a marking technology for materials. This makes it possible to trace the aging state in polymers as well as the powder mixture breakdown and to set process parameters that reduce scattering. Based on the methodical selection of modified polymers for use in SLS in part Ι [23] of this study, the aim of part ΙΙ was to experimentally validate the obtained result considering the available sinter material and SLS process technology. The focus was on the investigation of the impact of modified polymers on the intrinsic and extrinsic material properties, as well on the mechanical properties of the component, surface quality, and sinter density. Furthermore, the influence of the SLS process on the traceability of the modified polymers was determined by mass spectroscopy. The key conclusions can be outlined as follows:

- Within the scope of the investigation and the applied concentrations of the used modified polymer in the used polyamide 12 sinter material, the marking agent had no discernible influence on the material and component properties;
- The used modified polymer could be reliably detected in the printing powders as well as in the used powder and component down to a concentration of 1 ppm;
- No clear correlation was found between the concentration of the modified polymer and the determined intensity of the modified polymer in the used sinter material;
- A higher traceability of the modified polymer was obtained in the component than in the powder;
- Mixing parameters allowed a sufficient dispersion of the sinter material and master batch;
- Modified polymers were suitable as marking agents in the SLS process and allowed encoding of the used sinter material at the molecular level.

The suitability of the modified polymers evaluated in part Ι [23] of this study was confirmed in this work. Thus, the thermal stability of the modified polymers [33,34] was proven. The marked polyamide 12 sinter material can be evaluated as a substitution solution for the previously used sinter material within the considered concentration ranges of the modified polymer. Although the modified polymers had no measurable influence on the material and component properties, this needs further investigation. It is conceivable that the modified polymer is present in the material as a heterogeneous extraneous germ and influences the crystal formation as well as the morphology of the polymer [80]. Likewise, the modified polymer could accumulate in the amorphous phase of the polymer, since segregation and thus a segregation reaction occurs during crystallization [81]. The analysis of the morphology of the sinter material and master batch could reveal a possible influence of the modified polymer [73,82]. Similarly, the use of a high-purity sinter material could reveal a possible influence of the modified polymer on the morphology of the polymers.

For the implementation of the modified polymers as a marking technology in the SLS process, the extraction methodology has to be optimized to avoid the influence of carbon black particles on the measurement method and a quantification of the modified polymer. Based on a possible quantitative determination of the modified polymers, the degree of dispersion of the sinter material and master batch has to be determined. In addition, the effort of traceability has to be reduced and the analytical technique has to be integrated into the SLS process. In particular, the use of the desorption electrospray ionization–MS/MS analysis method in combination with modified polymers offers the potential of an in situ measurement of the marking agent [35,83,84,85]. Furthermore, the influence of aging and cyclic reuse as well as different process parameters on the traceability of the modified polymers has to be investigated. In addition, a possible migration of the modified polymer as a result of cyclic use must be investigated. A coding strategy can be developed based on the results. Therefore, part Ι [23] of this study has defined the tracer-based process optimization, which provides an analysis of the sinter material at defined stations in the SLS process and a targeted adjustment of the process parameters. Part Ι [23] of this study lists other potentially suitable marking technologies whose suitability also needs to be validated.

## Figures and Tables

**Table 1:** Description of the used specimen types in the investigated build job [37].

Type	Dimension	Number	Usage
Tensile bar XYZ	1BA ^1^	10	Tensile test
Tensile bar ZYX	1BA ^1^	10	Tensile test
Cube ^2^	15 × 15 × 15 mm	9	Sinter density, surface roughness
Cube ^3^	15 × 15 × 15 mm	8	Traceability

^1^ DIN EN ISO 527-2 [59]. ^2^ Cubes not framed in Figure 1. ^3^ Cubes framed with dotted line in Figure 1.

**Table 2:** Selected settings on the VL-550 optical coordinate measuring system [37].

Option	Selected Setting
Measuring method	Composition
Measuring mode	Manual
Resolution	Fine
Brightness	Auto (150)
Measuring view	Single view
Rotation method	Set angle
Degree	360°
Rotation segment	60°

**Table 3:** Selected settings on the MarSurf CM mobile confocal microscope [37].

Option	Selected Setting
Lens	800XS
Operating distance	1 mm
Brightness	100%
Exposure	40 ms
Reinforcement	1.5 dB
Measuring field	2.1401 mm × 2.1401 mm

**Table 4:** Breakdown of extraction methods for the samples under investigation.

Sample	Analyzed Quantity	Extraction Method	Duration in Ultrasonic Bath
Powder	0.5 g	Mixed with 10 mL ethanol Placed in ultrasonic bath at 40 °C Filtration with 22 μm filter Placed in a rotary vacuum evaporator Dilution with 2 mL methanol and 3 mmol ammonium acetate	30 min
Component	2 cubes ^1^ (3.3 g/cube)		60 min

^1^ Cubes listed in Table 1 for analyzing the traceability.

**Table 5:** Investigated material properties for the used polyamide 12 sinter material and master batch based on the used polyamide 12 sinter material and the modified polymer.

Properties	Sinter Material	Master Batch
D10 (μm)	28.2	28.7
D50 (μm)	66.3	65.2
D90 (μm)	100.0	99.0
Sphericity (-)	0.838	0.841
Aspect ratio (-)	0.710	0.712
Bulk density (g/cm^3^)	0.391 ± 0.003	0.390 ± 0.003
Tap density (g/cm^3^)	0.503 ± 0.003	0.502 ± 0.002
Crystallization temperature (°C)	157.73	157.51
Melting temperature (°C)	181.92	181.61
MFR (g/10 min)	14.67 ± 1.14	14.45 ± 0.83

## Data Availability

The raw/processed data required to reproduce these findings cannot be shared at this time as the data also form part of an ongoing study.
